# XGBoost-based ground motion model for constant-strength inelastic acceleration response spectra

**DOI:** 10.1038/s41598-026-46656-x

**Published:** 2026-04-02

**Authors:** Yuxuan Gong, Jiayue Zhao

**Affiliations:** 1 College of Construction Engineering, Jilin University, Changchun, 130026 China; 2 Key Lab of Structures Dynamic Behavior and Control of the Ministry of Education, Harbin Institute of Technology, Harbin, 150090 China

**Keywords:** Ground motion model, Inelastic response, Constant-strength response spectra, Inelastic spectral acceleration, Interpretability machine learning, Engineering, Natural hazards, Solid Earth sciences

## Abstract

Ground motion models (GMMs) are a fundamental component of probabilistic seismic hazard analysis (PSHA) and play a critical role in guiding structural seismic design by predicting ground motion parameters under specified earthquake scenarios. Since most structures enter a nonlinear state under the excitation of strong ground motions in big earthquake, the use of inelastic response spectra (IRS) for PSHA, structural seismic design and performance evaluation is considered more appropriate. In this study, the mixed-effects constant-strength inelastic spectral acceleration (ISA) prediction model was established by using 15,512 records from 171 earthquake events in the NGA-West2 database based on the XGBoost algorithm. The input parameters included moment magnitude (*M*_w_), Joyner-Boore distance (*R*_JB_), source parameters (Strike, T_plunge_, P_plunge_, Dip, and Rake angle), average shear-wave velocity up to 30 m depth (*V*_S30_), depth to the top of the rupture (*Z*_tor_), depth to the sedimentary layer with shear-wave velocity of 1 km/s (*Z*_1_), fault type (*F*_t_), as well as structural parameters (period *T* and strength reduction factor *R*_y_). To ensure the robustness of the proposed model, both within-event and between-event residual were analyzed to confirm its reliability and unbiasedness. Finally, the feature interpretability analysis was performed using permutation importance and SHAP methods, and the prediction accuracy was compared with the results of traditional GMM equation. The results demonstrate that the proposed machine learning model achieves high performance in predicting ISA, with period *T*, magnitude *M*_w_, and distance *R*_JB_ identified as the most influential features.

## Introduction

Ground motion models (GMMs) are an essential component of probabilistic seismic hazard analysis (PSHA), providing quantitative tools for estimating ground motion intensities (e.g., peak ground acceleration PGA, peak ground velocity PGV, peak ground displacement PGD) under different seismic sources, propagation paths, and site conditions. They have been widely applied in the development of seismic hazard maps, the formulation of seismic design codes, and the seismic safety assessment of critical infrastructure^1–3^. The advancement of GMM has not only enhanced the scientific basis and rationality of seismic design for structures but also provided vital support for seismic risk assessment, resilient urban development, and earthquake insurance pricing.

Most GMMs are derived from empirical regression analyses of historical ground motion records, where the regression equations rely on predefined linear or nonlinear functional forms. Boore and Stewart et al.^4,5^, based on the NGA-West2 database, proposed ground motion prediction equations (GMPEs) for estimating the median and standard deviation of intensity measures (IMs) for the average horizontal and vertical components of shallow crustal earthquakes in active tectonic regions. Similarly, Campbell et al.^6–9^, also using the NGA-West2 database, developed GMPEs for various IMs, such as Fourier amplitude spectra, by employing fixed-effects and mixed-effects regression analyses. However, this approach inherently constrains the expressive capacity of the models, as it still lacks a comprehensive consideration of multiple factors and struggles to capture the complex nonlinear interactions among source, path, and site effects. In particular, under strong ground motions or atypical site conditions, empirically based GMPEs often exhibit limited predictive accuracy and restricted applicability. Moreover, embedding additional complex parameters within a predefined functional form remains a significant challenge.

With the advancement of computational science, machine learning has been increasingly applied to the development of GMMs, opening new avenues for GMM^10^. Compared with traditional GMPEs, machine learning-based GMMs can avoid prior assumptions about functional forms and instead directly learn the complex mappings between inputs and outputs from data^11–13^. Existing studies have demonstrated that machine learning-based GMPEs outperform traditional empirical models in both predictive accuracy and applicability, highlighting their strong potential. Hu et al.^14^ employed support vector regression (SVR) to develop a GMM for the Kanto region of Japan, incorporating Arias intensity, cumulative absolute velocity (CAV), and effective duration as IMs. Sedaghati et al.^15^ applied a weighted ensemble approach to combine four machine learning models into a single framework for predicting various ground motion IMs. Ji et al.^16–18^ developed GMMs for multiple IMs using refined second-order neural networks, based on both horizontal and vertical recordings from the NGA-West2 database. Surendra et al.^19^ compared the performance of five machine learning algorithms in predicting PGA and PGV from strong-motion data in New Zealand, and further employed SHAP analysis to evaluate the relative importance of input features. SVR has also been successfully used to establish nonlinear relationships between engineering ground motion parameters and macro seismic intensity, demonstrating clear advantages over traditional linear regression approaches in intensity prediction^20^. Nevertheless, most of these studies remain confined to the level of ground motion parameters and fail to adequately capture the actual structural responses under seismic loading.

The response spectra serve as a bridge link between ground motions and structural responses, playing a critical role in transforming complex ground motion records into forms applicable for engineering seismic design and performance evaluation. Response spectra prediction models can directly estimate the maximum structural responses at different periods based on ground motion information, making them a central tool in engineering seismic design codes and performance evaluations. Neelamraju et al.^21^ employed conditional generative adversarial networks (C-GAN), incorporating physical characteristics of source, path, and site features into the adversarial training between generator and discriminator, and proposed a GMM for 5% damped acceleration response spectra (SA) based on the NGA-West2 database. Sreenath et al.^22^ applied transfer learning with XGBoost by leveraging a pre-trained model on global crustal data to develop a GMM for response spectra in the Himalayan region. Mohammadi et al.^23^ employed artificial neural networks (ANN) and XGBoost to predict PGA, PGV, and pseudo-spectral acceleration (PSA) from a Turkish dataset, and further developed an online platform for practical applications. In parallel, deep learning models have been developed to directly predict nonlinear structural responses of hysteretic systems, including strength degradation and pinching effects, by learning from nonlinear time-history analyses, demonstrating the capability of machine learning to capture complex inelastic behavior beyond elastic spectral representations^24,25^. The aforementioned studies have primarily focused on the prediction and application of elastic response spectra, which assume that structures remain in the elastic range under seismic loading. This is necessary and satisfactory for the strength-based seismic design of the structures. However, under moderate to strong earthquakes, most structures may be put into the inelastic state, and their actual responses may be significantly different from the values predicted by elastic theory. Which means that the elastic response spectra cannot satisfy the evaluation of nonlinear structural performance evaluation.

Therefore, the inelastic response spectra (IRS), as an indicator that better reflects the actual seismic demand on structures, have gradually attracted increasing attention from researchers^26–28^. Bozorgnia et al.^29^ considered the correlation of inelastic spectral ordinates with earthquake magnitude (*M*_w_), site-to-source distance (*R*), faulting mechanism, local soil conditions, and basin effects, and developed a GMPE for constant-ductility displacement spectra. Cheng et al.^30^ constructed GMPEs for constant-ductility and constant-strength input energy spectra based on single-degree-of-freedom (SDOF) systems with four hysteretic models. Bahrampouri et al.^31^ proposed a GMM for inelastic displacement spectra using the NGA-West2 database and found that inelastic behavior reduces both intra-event and inter-event variability of response spectra, particularly in at the short period range. Dong et al.^32^ developed the inelastic displacement, velocity, and acceleration spectra for self-centering structures with flag-shaped hysteretic behavior subjected to near-fault pulse-like ground motions. Although IRS has been explored to some extent in theoretical and numerical studies, most existing prediction models still rely on simplified empirical formulas, which struggle to maintain high accuracy and generalization across diverse conditions. In contrast, machine learning methods have already demonstrated significant advantages in GMMs, offering new opportunities to develop more accurate machine learning-based prediction models for IRS. Hammal et al.^33^ employed ANN to predict the inelastic displacement spectra of SDOF systems with 5% damping, and the *M*_w_, source depth, *R*, average shear-wave velocity up to 30 m depth (*V*_S30_) strength yielding and SDOF period (*T*) are considered as the input parameters in their prediction model. Dwairi et al.^34,35^ further applied ANN models to estimate the inelastic displacement demands of SDOF structures located on both soft and firm soils, demonstrating the reliability of machine learning in predicting seismic responses. Meanwhile, machine learning has also been widely applied to rapid post-earthquake structural damage assessment^36,37^. However, these studies do not incorporate interpretability analysis, which makes it difficult to be understanded the internal decision-making process of the models and to identify the relative importance of different input parameters.

As an important and typical form of the IRS, the inelastic acceleration response spectra are widely used in structural seismic design verification, seismic performance evaluation, seismic hazard analysis, etc. For a prescribed strength reduction factor, the spectral ordinate of the constant-strength form of the IRS is defined as the constant-strength inelastic spectral acceleration (ISA). In this study, an XGBoost-based approach is developed to predict ISA using the NGA-West2 database, leveraging the nonlinear learning capability of machine learning to improve prediction accuracy and generalization. Interpretable machine learning techniques are further incorporated to quantify the contributions of source, path, site, and SDOF system parameters to the predicted ISA. The proposed model is validated with widely used traditional GMM prediction model. From an engineering perspective, the proposed ISA-based model can be directly integrated into PSHA to construct IRS hazard curves and uniform hazard spectra, extending conventional elastic GMMs to the nonlinear domain. In addition, the predicted inelastic spectral demands can be applied in performance-based seismic design (PBSD) and assessment to estimate nonlinear structural responses without relying on elastic-to-inelastic conversion factors, and may further serve as a data-driven basis for developing inelastic design spectra in seismic codes. By achieving both high predictive accuracy and interpretability, this approach not only addresses the limitations of conventional IRS models but also provides a foundation for PBSD and rapid risk assessment.

## Ground motion selection and ISA computation

This section describes the process of dataset construction, including the selection of ground motion records, the hysteretic models employed for computing inelastic responses, and the computation of method of the ISA.

### Ground motion records information

**Fig. 1:**
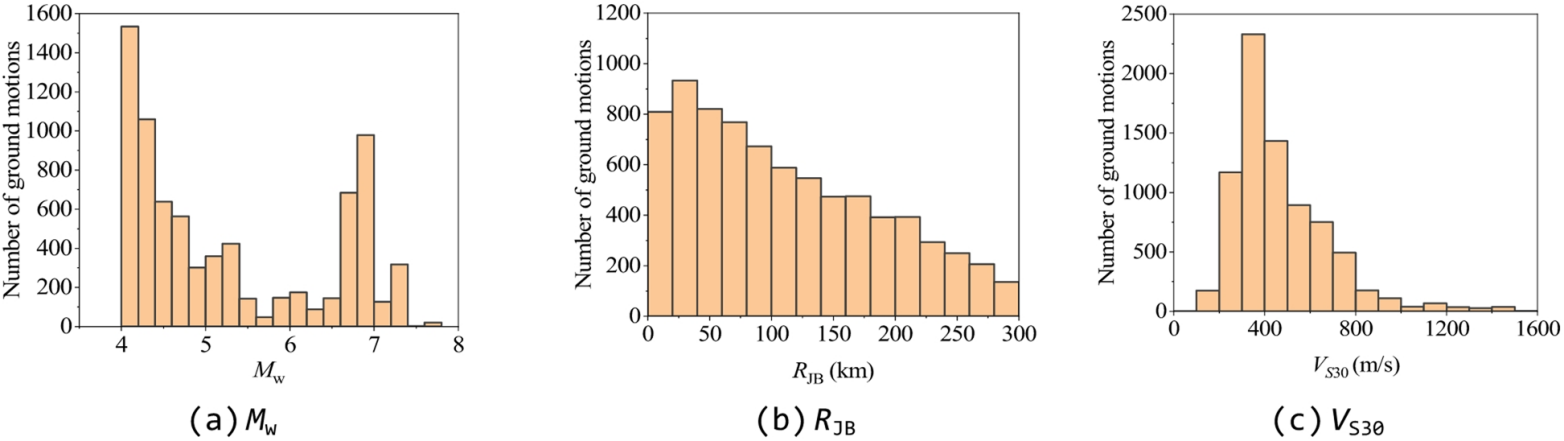
Histograms of the number of selected ground motions.

**Fig. 2:**
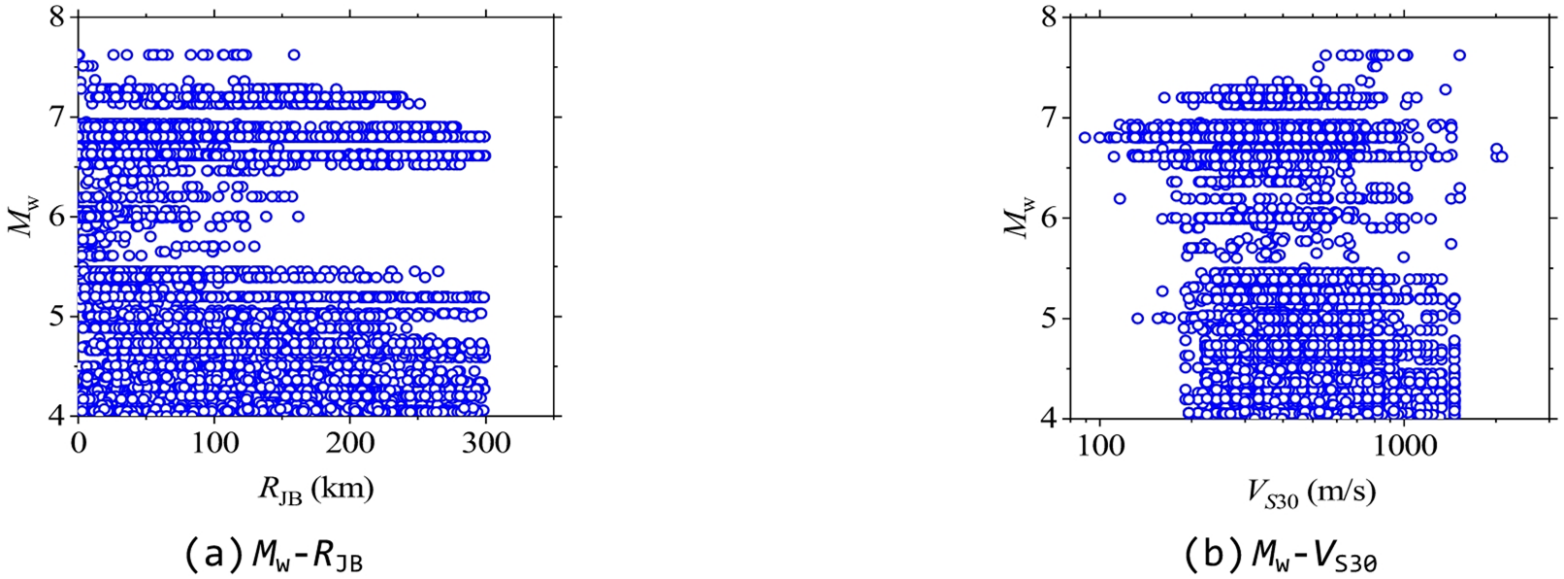
Distributions of *M*_w_-*R*_JB_ and *M*_w_-*V*_S30_ pairs for the selected ground motions.

The ground motion records used in this study for calculating ISA were selected from the NGA-West2 database^38^ according to the following criteria: (1) metadata on magnitude, distance, and site information must be available; (2) *V*_S30_ estimates for seismic stations must be accessible; (3) *M*_w_ is between 4.0 and 8.0, and the Joyner-Boore distance (*R*_JB_) is less than 300 km. Based on these criteria, a total of 15,512 ground motion records (single horizontal component) from 171 earthquake events were selected. The distribution of *M*_w_, *R*_JB_, and *V*_S30_ of these ground motion records is shown in Fig. 1, and the distribution for these ground motion records in *M*_w_-*R*_JB_ and *M*_w_-*V*_S30_ coordinates of the entire dataset is shown in Fig. 2.

### ISA computation approach

The ISA, as one of the IRS, is widely used in structural seismic design verification and performance evaluation. In general, the ISA is computed using an elasto-plastic SDOF system and represents the maximum response of a structure with a given yield strength or ductility capacity under a specified ground motion input. When the strength reduction factor (*R*_y_) is kept constant, the resulting spectra are referred to as the constant-strength IRS. The *R*_y_ is defined as the ratio of the maximum displacement of the corresponding elastic system to the yield displacement^30^.

**Fig. 3:**
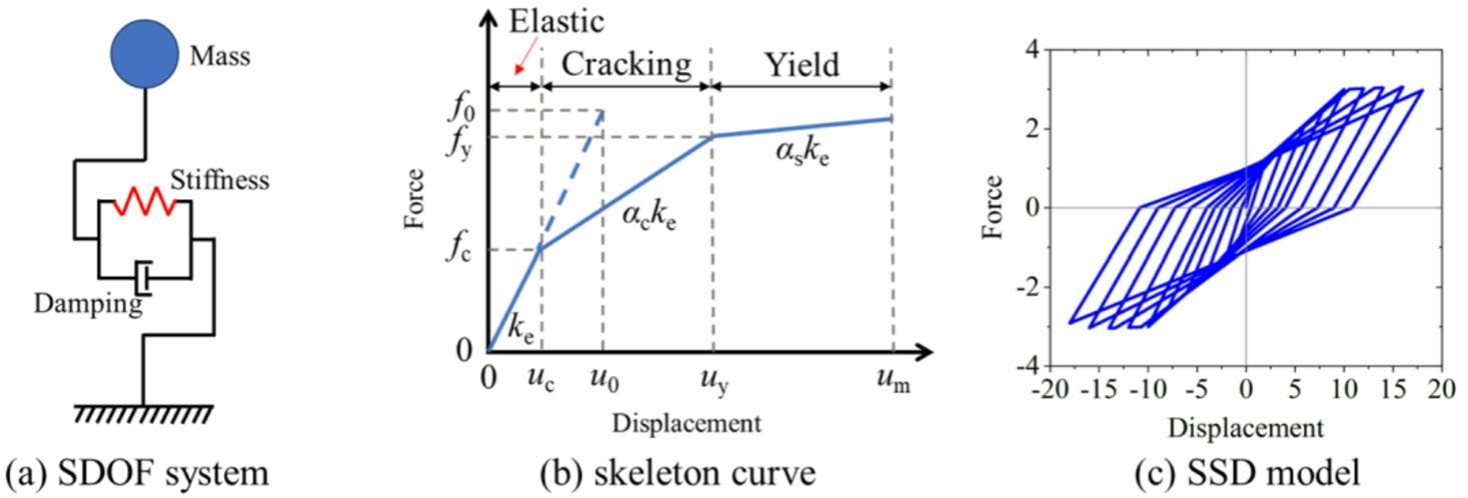
SDOF system and hysteretic models.

In this study, an SDOF system was established based on the trilinear restoring force model proposed by Molazadeh et al.^39^. This model is a trilinear strength-stiffness degradation model (SSD), in which the cracking strength is set as one-third of the yield strength, the post-yield stiffness is 2% of the elastic stiffness, and unloading stiffness degradation is considered. The hysteretic model is widely used in the seismic response simulation of the Reinforced Concrete (RC) structures. Figure 3 presents the SDOF structure system, the backbone curve of the hysteretic model, and the hysteresis loops. Here, *k*_e_ denotes the elastic stiffness; *u*_c_ and *f*_c_ are the cracking displacement and force; *u*_y_ and *f*_y_ are the yield displacement and force; *α*_c_ and *α*_s_ represent the first and second reduction factors of *k*_e_; *u*_m_ is the maximum inelastic displacement; and *u*_0_ and *f*_0_ correspond to the maximum displacement and force of the associated elastic system under the same ground motion. According to the definition of the strength reduction factor, *R*_y_ can be calculated as shown in Eq. (1):

1 $${R_y}={{{f_0}} \mathord{\left/ {\vphantom {{{f_0}} {{f_y}}}} \right. \kern-0pt} {{f_y}}}={{{u_0}} \mathord{\left/ {\vphantom {{{u_0}} {{u_y}}}} \right. \kern-0pt} {{u_y}}}$$

**Fig. 4:**
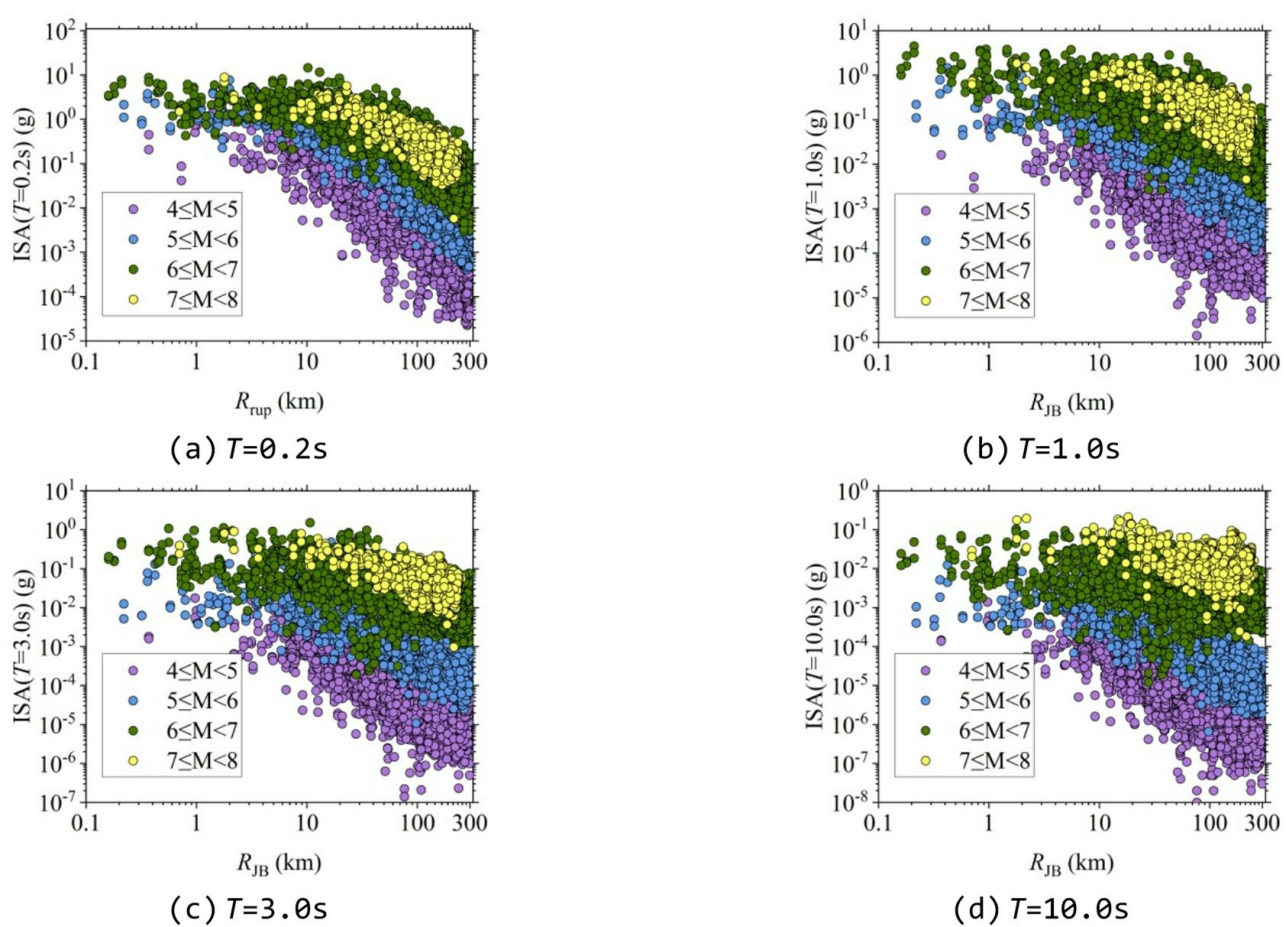
Distributions of ISA-*R*_JB_ pairs for the selected ground motions (*R*_y_=4).

To compute the ISA from the seismic response analysis, the SDOF structures were established in OpenSees using zero-length elements^40^. The uniaxial Hysteretic material model was created with the Hysteretic command, and the hysteretic model parameters were adopted following Zhao et al.^41^. Subsequently, SDOF models with different periods were constructed for specified *R*_y_. Since *R*_y_=1 corresponds to elastic structures, five inelastic models with *R*_y_ values ranging from 2 to 6 were developed. The structural periods were set identically to the 21 periods used in CB14^6^, ranging from 0.01 to 10 s, and a damping ratio of 0.05 was adopted. After establishing the SDOF structures, time-history analyses were conducted under the excitation of the selected ground motions. The maximum response of each SDOF structure under each ground motion was extracted to obtain the ISA, resulting in a total of 1,954,512 data sets. Figure 4 shows the distribution of the ISA with respect to *R*_JB_ for some typical periods when *R*_y_=4.

## Methodology

This section provides an overview of the techniques used to generate the GMMs for ISA. The input parameters for model development, the machine learning algorithms employed, and the hyperparameter optimization methods are introduced. Finally, the metrics used to evaluate model performance are presented.

### Input features

Thirteen parameters were selected as inputs for the machine learning model, including *M*_w_, *R*_JB_, source parameters (Strike, T_plunge_, P_plunge_, Dip, and Rake angle), average shear-wave velocity up to 30 m depth (*V*_S30_), depth to the top of the rupture plane (*Z*_tor_), depth of the sediment layer with a shear wave velocity of 1 km/s (*Z*_1_), fault type (normal, reverse, or strike-slip), and structural parameters (period *T* and *R*_y_). The output corresponds to the natural logarithm of ISA.

### Mixed-effects model

Mixed-effects models are particularly suitable for datasets involving repeated measurements, where variability arises from multiple hierarchical sources. In such models, the total residual is decomposed into fixed and random components. In this study, ground motion records are obtained from multiple earthquakes and stations, covering a wide range of site conditions and source-to-site distances. It is well recognized that ground motion variability is substantial not only between different earthquake events but also among records within the same event, which motivates the decomposition of total residuals into distinct components^42^.

To account for this hierarchical variability, a mixed-effects framework is incorporated into the proposed machine-learning-based GMM. The fixed effects are learned by a machine learning model that captures the systematic dependence of ground motion IM on the predictor variables, whereas the random event-specific effects are quantified through post-hoc residual decomposition, following the implementation procedure described in Wang et al.^43^. This approach preserves the residual structure and physical interpretability of traditional ground motion models, while benefiting from the flexibility of machine learning in representing complex nonlinear relationships.

### XGBoost algorithm

XGBoost (eXtreme Gradient Boosting) is an efficient ensemble learning algorithm based on gradient boosting trees^44^, whose core main idea is to iteratively construct and integrate multiple weak learners to improve overall predictive performance. Moreover, XGBoost can capture complex multi-variable interactions (e.g., magnitude–distance–period coupling) that are difficult to represent using predefined functional forms in conventional GMMs^22,23^. These relationships are learned directly from the data without imposing an explicit parametric structure.

**Fig. 5:**
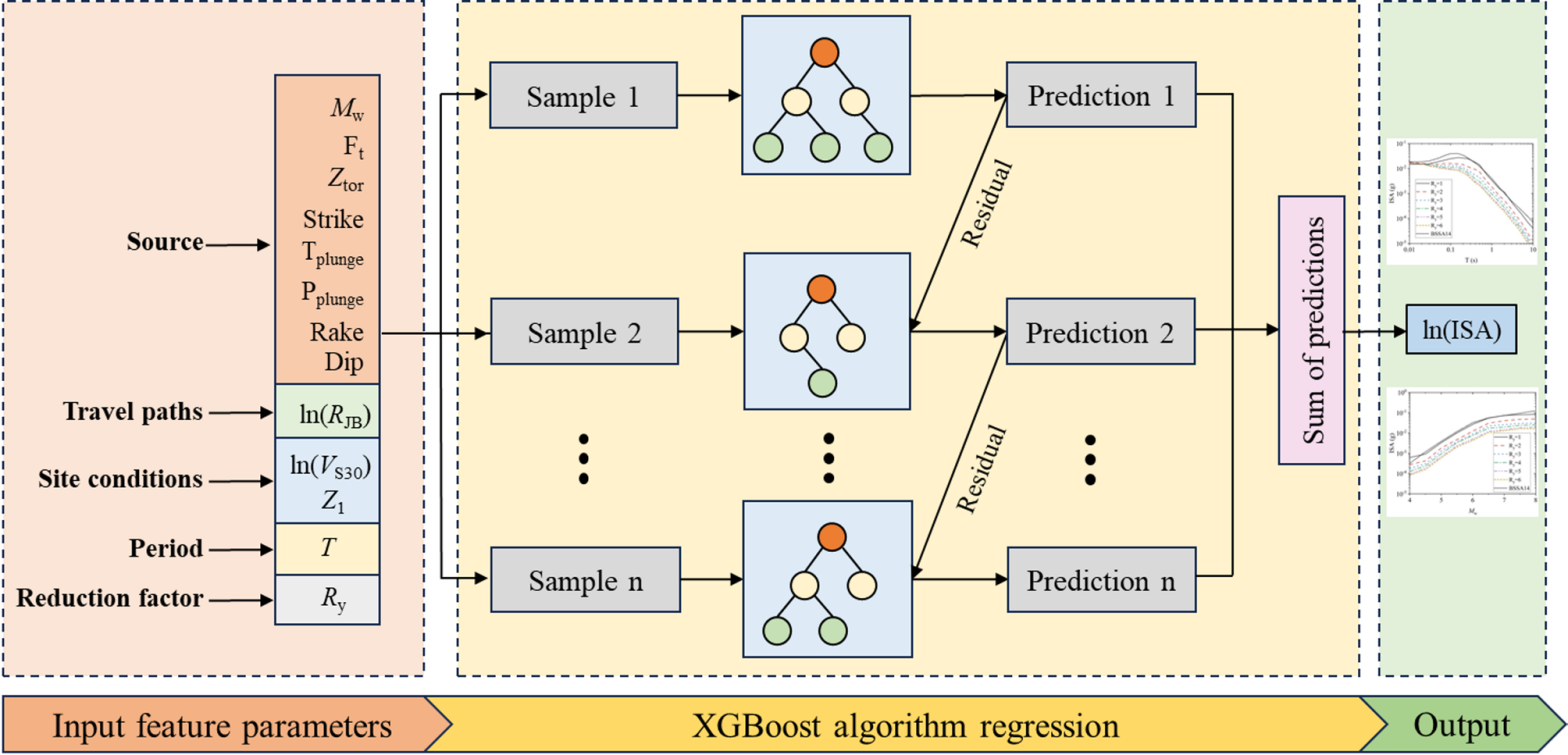
The flowchart of the analysis and the XGBoost Structure.

Figure 5 illustrates the structure of the XGBoost method and the flowchart of the whole work in this study. In the XGBoost model, a tree is trained using a randomly selected subset of data to predict the target, and the residuals from this prediction are then used to train the next tree. This process is repeated iteratively: the predictions from the new tree are added to the existing model, the updated model computes smaller residuals, and these residuals are used to train subsequent trees. The iteration continues until the preset number of trees is reached or the residuals become sufficiently small. The final output can be obtained by summing the predictions of all trees.

### Hyperparameter optimization method

In this study, the Optuna library^45^ was employed for hyperparameter optimization. By default, Optuna uses the Tree-structured Parzen Estimator (TPE) algorithm, a Bayesian optimization method that adaptively models the probability distributions of promising and less promising hyperparameter regions. This approach enables the search process to more efficiently focus on hyperparameter combinations that are likely to yield better model performance. The hyperparameter search space in this study included learning rate, tree depth, subsample ratio, column sampling ratio, minimum child weight, gamma, and regularization parameters. The optimization objective was to minimize the mean squared error on the validation set, and the search was conducted 30 trials.

### Interpretability of machine learning

To enhance the transparency of model predictions, this study employed both Permutation Importance^46^ and SHapley Additive exPlanations (SHAP)^47^ for interpretability analysis. Permutation Importance is a model-gnostic feature importance evaluation method, which quantifies the importance of a feature by randomly shuffling its values and observing the resulting change in model performance. If shuffling a feature leads to a significant decrease in model performance, it indicates a high contribution of that feature to the predictions. On the contrary, a minor change suggests a limited role. Unlike metrics based on internal model structures, such as split gains in tree models, Permutation Importance directly relies on changes in predictive performance, allowing feature contributions to be compared across different models and reducing the influence of model structure bias.

On the other hand, SHapley Additive exPlanations (SHAP)^47^, based on Shapley values from game theory, interprets model outputs by quantifying the marginal contribution of each input feature to the prediction. The method sets a baseline and then evaluates the difference between predictions under various feature combinations and the baseline to calculate the contribution of each individual feature. By combining these two approaches, the key input variables’ importance can be revealed, and the positive and negative effects of features on predictions are simultaneously illustrated. Thereby, the interpretability and credibility of the model can be enhanced.

### Evaluation of the model performance indicators

The most widely used three statistical metrics to evaluate the performance of regression, R², RMSE, and MAE, were adopted to evaluate model performance in this study. These metrics are calculated as shown from Eq. (2) to Eq. (4):

2 $${R^2} = 1 - \frac{{\mathop \sum \limits_{i = 1}^n {{\left( {{{\hat y}_i} - {y_i}} \right)}^2}}}{{\mathop \sum \limits_{i = 1}^n {{\left( {\bar y - {y_i}} \right)}^2}}}$$

3 $$RMSE = \sqrt {\frac{1}{n}\mathop \sum \limits_{i = 1}^n {{\left( {{y_i} - {{\hat y}_i}} \right)}^2}}$$

4 $$MAE = \frac{1}{n}\mathop \sum \limits_{i = 1}^n \left| {{y_i} - {{\hat y}_i}} \right|$$

Here, *n* denotes the number of samples, ${y_i}$ is the calculated value (ISA value calculated from SDOF time-history analysis), ${\hat {y}_i}$ is the predicted value (ISA value predicted from the model), and $\bar {y}$ is the arithmetic mean of ${y_i}$, respectively. Among these performance metrics, *R*² quantifies the proportion of variance in the response variable that can be predicted by the explanatory variables. The error-related metric, *RMSE*, represents the average distance between the calculated and predicted ISA value. Moreover, *MAE* represents the mean absolute difference between the calculated and predicted ISA value. According to the definitions of the three metrics, the model performance improves as *R*^2^ increases while *RMSE* and *MAE* decrease.

Residuals, commonly decomposed into between-event and within-event types in ground motion prediction modeling, serve as essential metrics for evaluating the accuracy and reliability of the model. The computation of residuals are shown from Eq. (5) to Eq. (6):

5 $$\ln {({\mathrm{ISA}})_{ij}}=\overline {{\ln {{({\mathrm{ISA}})}_{ij}}}} +{\eta _i}+{\varepsilon _{ij}}$$

6 $${\eta _i} = \frac{1}{{{n_i}}}\sum\limits_{j = 1}^{{n_i}} {{{{\Delta }}_{ij}}}$$

Here, $\ln {({\mathrm{ISA}})_{ij}}$ and $\overline {{\ln {{({\mathrm{ISA}})}_{ij}}}}$ denote the actual and predicted ISA values of the *j*th record from the *i*th earthquake respectively, and ${{{\boldsymbol{\Delta}}}_{ij}}$ represents the total residual; ${\eta _i}$ represents the between-event residuals, and is a random variable with a standard deviation of $\tau$; ${\varepsilon _{ij}}$ denotes the within-event residuals, and is also a variable with a standard deviation of $\varphi$. The within-event residual represents the difference between predicted and actual values for all ground motion records, while the between-event residual reflects the residuals across individual earthquake events. Under the conventional assumption that the between-event and within-event residuals are uncorrelated, the total standard deviation $\sigma$ can be calculated as:

7 $$\sigma =\sqrt {{\tau ^2}+{\varphi ^2}}$$

## Results analysis

The constructed ISA database was randomly divided into training, validation, and test sets with a ratio of 8:1:1 at the earthquake event level. Specifically, all records from the same earthquake event were assigned exclusively to a single subset to avoid potential data leakage. The training and validation sets were used for model development and hyperparameter tuning, while the test set was reserved for an independent evaluation of the model’s predictive performance.

### Comparison with other machine learning models

The predictive performance of the proposed mixed-effects machine-learning-based GMM is compared with three widely used tree-based machine learning models, including XGBoost, LightGBM, and Random Forest. All models are trained using the same input features and identical training–validation–test splits to ensure a fair comparison.

As shown in Table 1, XGBoost and LightGBM exhibit nearly identical predictive performance, with *R*² values of 0.9238 and 0.9237, respectively, and comparable RMSE and MAE values, indicating that both gradient boosting models effectively capture the nonlinear relationships in the dataset. In contrast, the Random Forest model shows noticeably lower performance, with a reduced R² and larger error metrics, suggesting comparatively weaker predictive capability. Beyond conventional accuracy metrics, residual-based statistics further highlight model performance. Given the comparable performance of XGBoost and LightGBM, XGBoost is adopted in this study as the baseline machine learning model for subsequent development and integration within the proposed mixed-effects framework.

**Table 1:** Performance indicators (in Logarithmic Form) of test set for different models.

Model	*R* ^2^	RMSE	MAE	Mean residual	τ	φ	σ
XGBoost	0.9238	0.9300	0.7284	0.2065	0.6648	0.7370	0.9926
LightGBM	0.9237	0.9305	0.7350	0.1666	0.7262	0.7257	1.0267
Random Forest	0.8930	1.1023	0.8533	0.2696	0.7637	0.8905	1.1731

### Interpretability of the XGBoost-based GMM

**Fig. 6:**
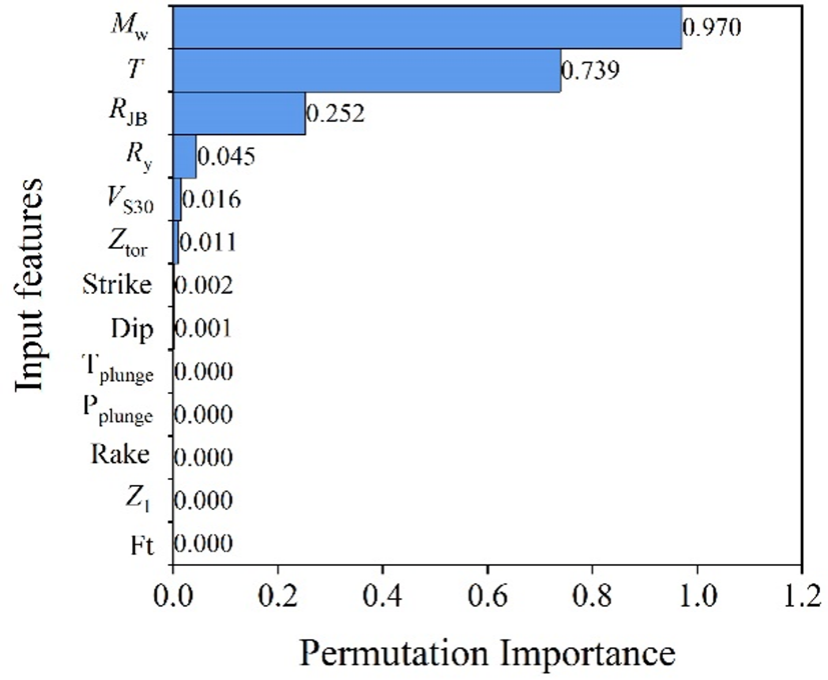
Feature importance ranking based on Permutation Importance.

The interpretability of the proposed XGBoost-based ISA prediction model is analyzed using the previously described Permutation Importance and SHAP methods. Figure 6 shows the feature importance ranking based on Permutation Importance. The permutation importance values correspond to the average reduction in prediction accuracy induced by feature permutation and are therefore scale-dependent and comparable only across predictors within the same model. It can be observed that model predictions primarily depend on three features: *M*_w_, *T* and *R*_JB_. Specifically, *M*_w_ exhibits the highest importance, followed closely by *T* and *R*_JB_, indicating that earthquake magnitude, SDOF structural period, and *R*_JB_ distance exert the strongest influence on the predicted ISA. *R*_y_ shows moderate importance, indicating that structural nonlinear characteristics also have a noticeable, though secondary, effect on ISA. In contrast, site and fault parameters such as *V*_S30_ and *Z*_tor_ have lower importance, serving only as minor adjustments, while features like Rake and F_t_ contribute almost negligibly. Overall, these results align well with empirical trends observed in traditional GMPEs, reaffirming that *M*_w_, *T*, and *R*_JB_ remain the dominant predictors of ground motion intensity^4,6^, while *R*_y_ and other secondary parameters act as modifiers reflecting site and structural effects.

**Fig. 7:**
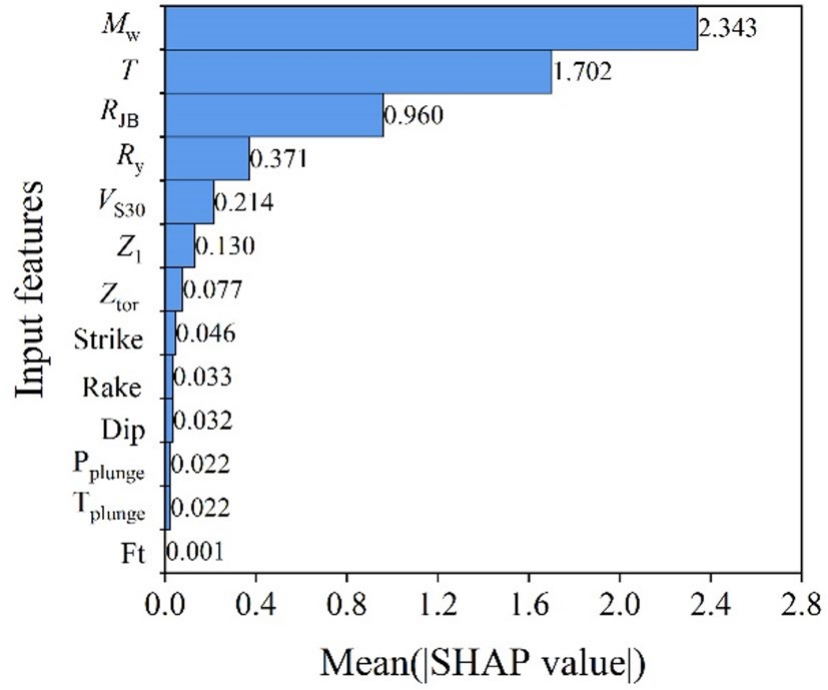
Global SHAP value rankings.

**Fig. 8:**
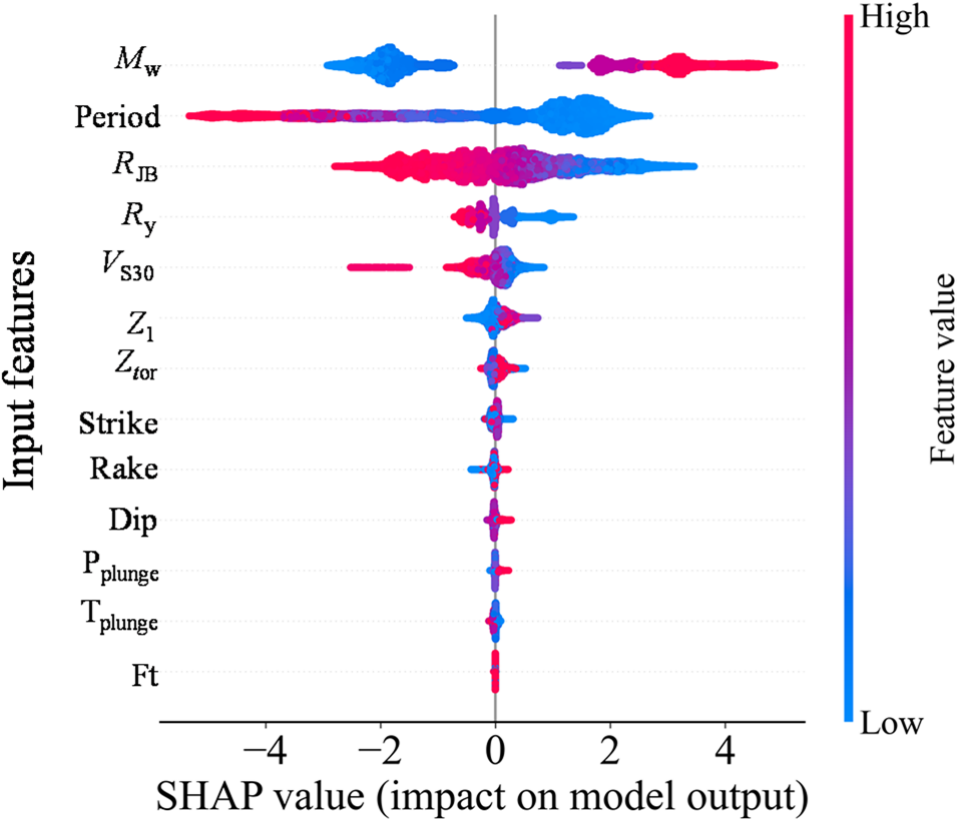
Summary of the SHAP value rankings.

Figure 7 presents the SHAP-based feature importance results. The horizontal axis represents the mean absolute SHAP value, indicating the importance of each input feature to model predictions, and larger values correspond to a greater overall contribution to the prediction. Figure 8 is the SHAP summary plot. The horizontal axis represents SHAP values, indicating the positive or negative effect of each feature on model output. The vertical axis lists input features, and the color encodes the feature values (red for high, blue for low), illustrating the direction and magnitude of each feature’s influence across different values.

**Fig. 9:**
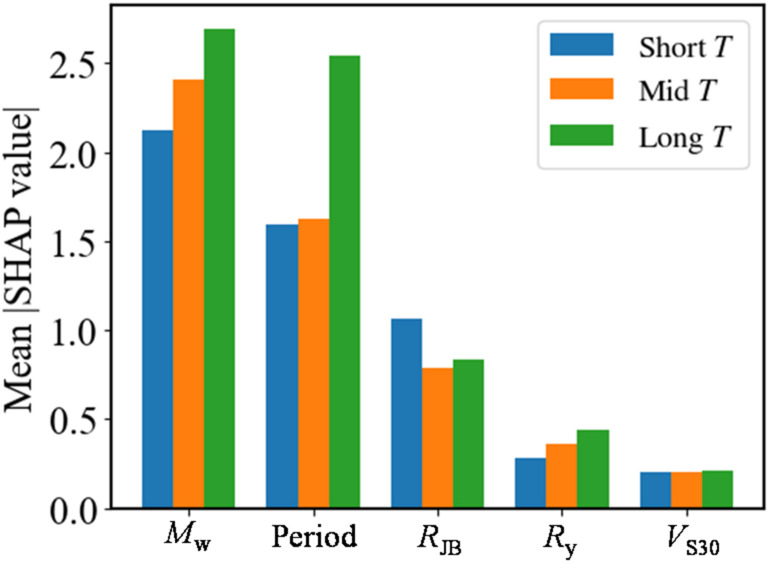
T-dependent SHAP importance.

Figure 9-Fig. 11 further extend the SHAP analysis by examining period-dependent feature importance, feature interaction effects, and variability across different intensity levels, respectively. To facilitate this analysis, the dataset is subdivided according to *T* (short: *T* ≤ 0.2s; medium: 0.2 < *T* ≤ 1.0s; long: *T >* 1.0s) and ISA level (low, medium, high based on the data distribution). For clarity, only the top five most influential features are displayed.

**Fig. 10:**
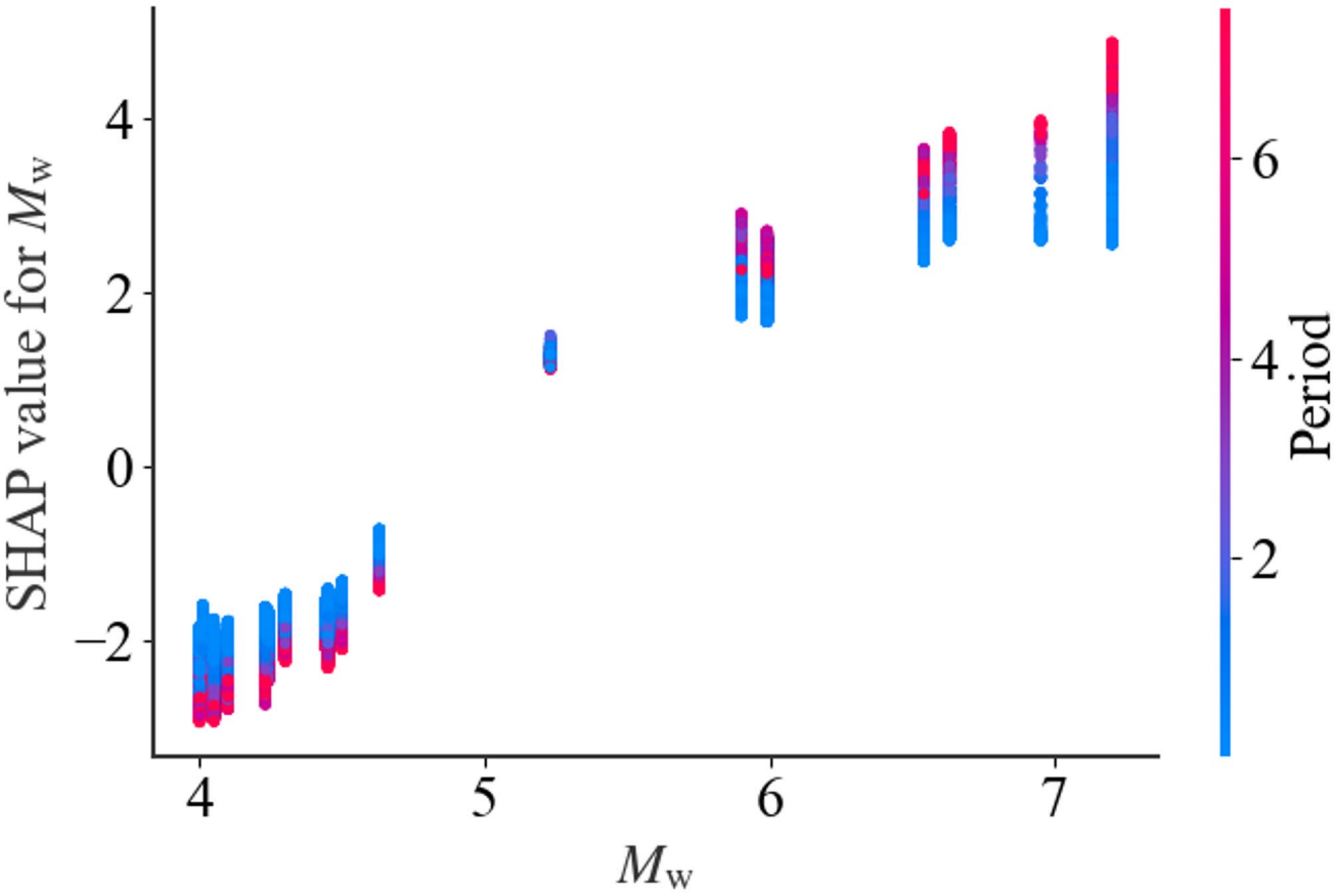
SHAP dependence of *M*_w_.

**Fig. 11:**
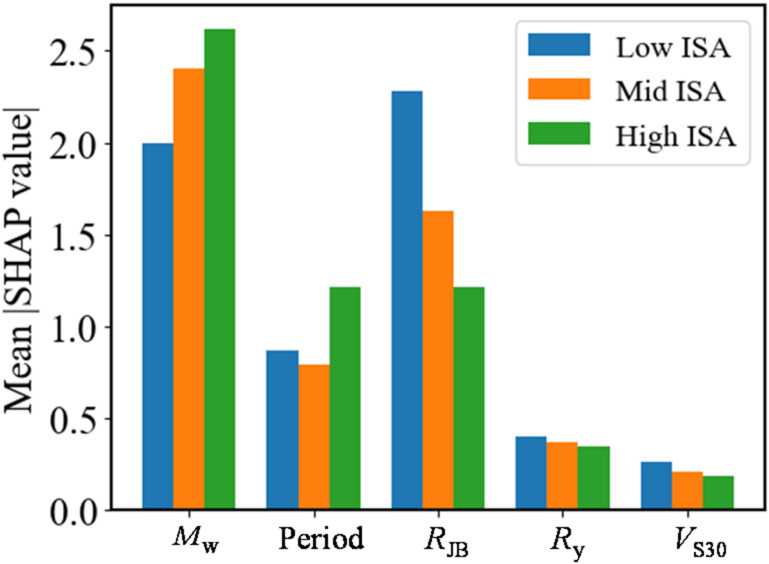
SHAP importance by ISA level.

The SHAP analysis further validates the dominance of *M*_w_, *T*, and *R*_JB_ in governing the model predictions. As shown in Fig. 7, these three variables exhibit substantially higher mean absolute SHAP values than the others, indicating that they are the principal determinants of the predicted ISA. The SHAP distribution also clarifies the directional effects of each feature: larger *M*_w_ and smaller *R*_JB_ correspond to positive SHAP values, thereby increasing the predicted ISA, while lower magnitudes and larger distances reduce it. Likewise, shorter structural periods lead to higher predicted spectral accelerations which is consistent with the typical shape of response spectra. The moderate influence of *R*_y_ indicates that higher *R*_y_ values (representing stronger inelastic behavior) tend to reduce the predicted ISA. Meanwhile, the effect of *V*_S30_ follows expected site amplification trends, where lower *V*_S30_ amplifies ISA. Which is consistent with the existing knowledge that the soft site could amplify the earthquake ground motion. Other parameters exhibit limited SHAP impacts, confirming their secondary roles.

The SHAP analysis across different periods and ISA levels reveals several clear patterns. As shown in Fig. 9, the mean SHAP values generally increase with spectral period, with long-period responses exhibiting the largest contributions, indicating that the model relies more heavily on key predictors for longer-period SDOF systems. Figure 10 highlights the effect of earthquake magnitude *M*_w_, which has a strong positive impact on model predictions and interacts with spectral period. Figure 11 shows that high-intensity events correspond to larger SHAP values for dominant features, while low-intensity events contribute less. These results demonstrate that both period and intensity influence the relative importance of predictors, and the model captures these nonlinear relationships effectively.

Based on the combined evidence from SHAP analysis and permutation importance results, *M*_w_, T, *R*_JB_, *R*_y_, and *V*_S30_ are ultimately selected as the input features for training the final model, as they capture the dominant source, path, site, and inelastic behavior effects while maintaining model parsimony and practical applicability. Overall, the SHAP analysis not only supports the findings of the permutation importance evaluation but also provides additional insight into both the magnitude and direction of each feature’s contribution to the model output.

### Hyperparameter optimization results

A model including the predictors *M*_w_, T, *R*_JB_, *R*_y_, and *V*_S30_ was constructed for ISA prediction, and hyperparameter optimization was performed using the Optuna library. The hyperparameter search space, the final selected hyperparameters, and their relative importance are summarized in Table 2. It can be observed that among all hyperparameters, the learning_rate is the most important for the training model while the importance of colsample_bytree and reg_lambda is almost negligible.

**Table 2:** Optimal parameters used in XGBoost.

Hyperparameter	Short explanation	Parameter range	Determined optimal parameter	Hyperparameter importance
learning_rate	n_estimators	0.02–0.20	0.026	0.79
max_depth	maximum depth of trees	3–8	8	0.04
subsample	subsample ratio of the training dataset	0.60–1.00.60.00	0.766	0.01
colsample_bytree	subsample ratio of column	0.60–1.00.60.00	0.631	< 0.01
min_child_weight	minimum child weight	5–30	28	0.09
gamma	controlling tree complexity	0–5	0.903	0.04
reg_alpha	L1 regularisation term on weights	0–0.1.1	0.000	0.01
reg_lambda	L2 regularisation term on weights	0–10	0.097	< 0.01

### Performance evaluation of model

**Fig. 12:**
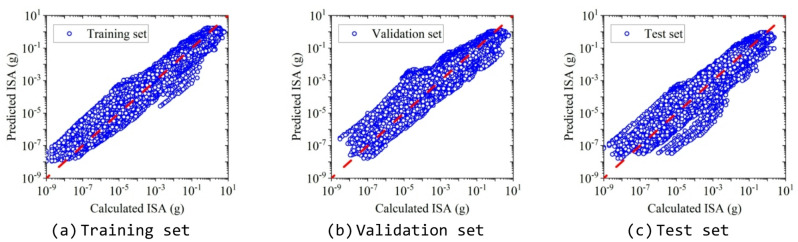
Distribution of the calculated and predicted ISA values.

The model trained with the optimized hyperparameters was adopted as the final model, and its performance was evaluated using the test set. Figure 12 presents the distributions of the calculated ISA versus the predicted ISA by the XGBoost model for the training, validation, and test sets. It can be seen that the scatter distributions of all three datasets are similar, indicating consistent predictive capability across datasets, which means that the XGBoost model achieved reliable predictions. To assess potential overfitting of the trained XGBoost model, three performance metrics (*R*², *RMSE*, and *MAE*) were computed for the training, validation, and test sets, as shown in Table 3. From which we can see that the proposed XGBoost model exhibits excellent predictive performance across all datasets. The *R*² exceeds 0.92 for the training, validation, and test datasets, indicating a strong agreement between the predicted and observed logarithmic ISA values. Moreover, the nearly identical performance on the validation and test datasets demonstrates the robustness and stability of the proposed model.

The performance metrics for different typical periods across the training, validation and test datasets are presented in Table 4. From which it can be seen that the proposed XGBoost model maintains high predictive performance across a wide range of vibration periods, with performance gradually improving as the period increases. In particular, medium- to long-period SDOF systems show the best predictive performance, while short-period responses exhibit slightly lower accuracy. Overall, the results indicate that the model effectively captures the nonlinear relationship between ground motion parameters and inelastic response, and generalizes well across different period ranges.

**Table 3:** Performance indicators (in Logarithmic Form) of different datasets.

Datasets	*R* ^2^	RMSE	MAE
Training	0.9741	0.5641	0.4408
Validation	0.9263	0.8271	0.6481
Test	0.9266	0.9128	0.7172

**Table 4:** Performance indicators (in Logarithmic Form) for ISAs with different periods.

ISA	Datasets	*R* ^2^	*RMSE*	*MAE*
ISA (*T*=0.2s)	Training	0.9436	0.6148	0.4817
	Validation	0.8677	0.8086	0.6198
	Test	0.8571	0.9209	0.7201
ISA (*T*=1.0s)	Training	0.9507	0.6269	0.4936
	Validation	0.8911	0.8385	0.6572
	Test	0.8837	0.9077	0.7189
ISA (*T*=3.0s)	Training	0.9613	0.6200	0.4867
	Validation	0.9172	0.8354	0.6758
	Test	0.9140	0.8912	0.7092
ISA (*T*=10.0s)	Training	0.9623	0.6170	0.4861
	Validation	0.9244	0.8232	0.6551
	Test	0.9372	0.8937	0.7229

### Residual analysis

**Fig. 13:**
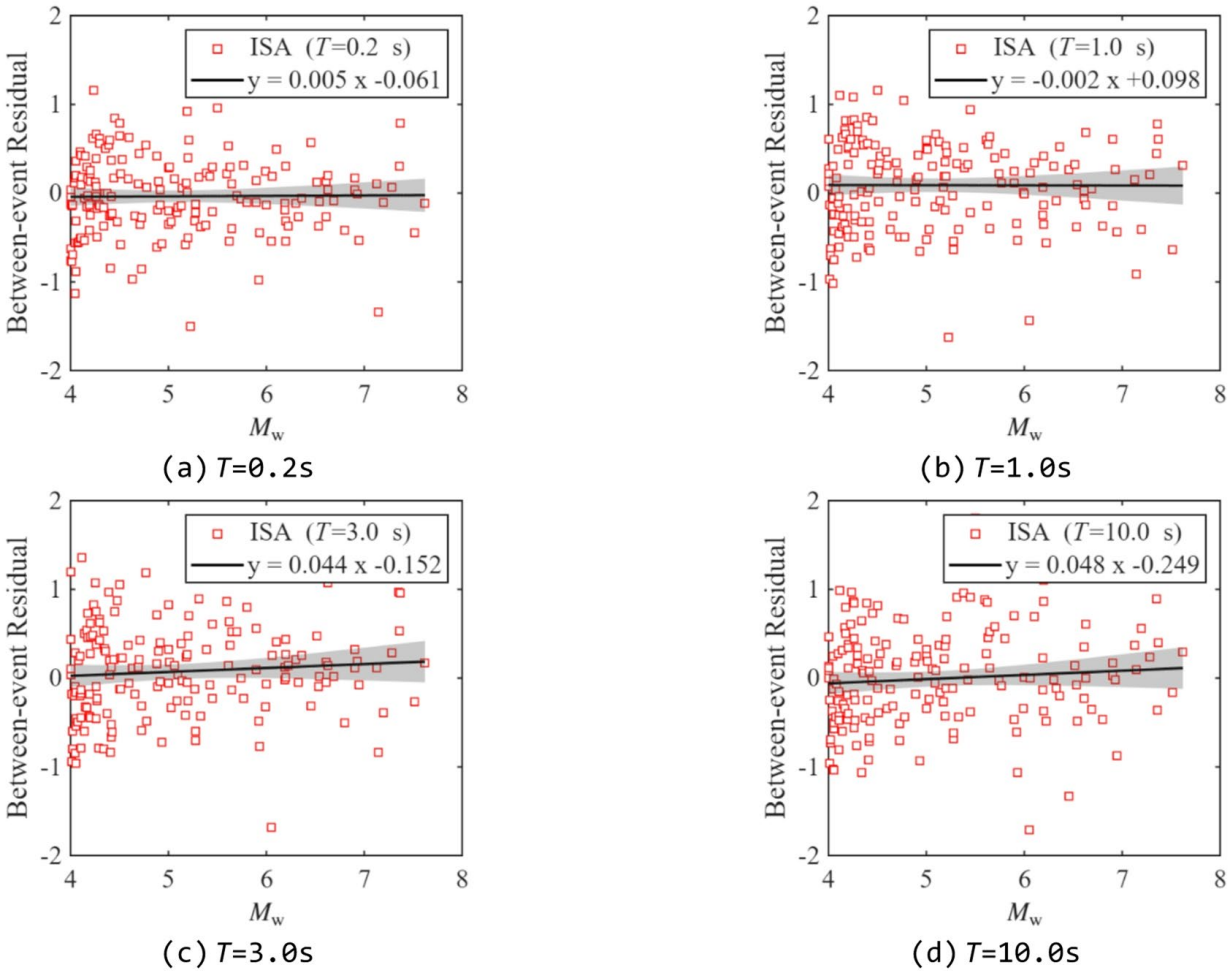
Distribution of between-event residuals of ISAs related to *M*_w_ (*R*_y_=4).

**Fig. 14:**
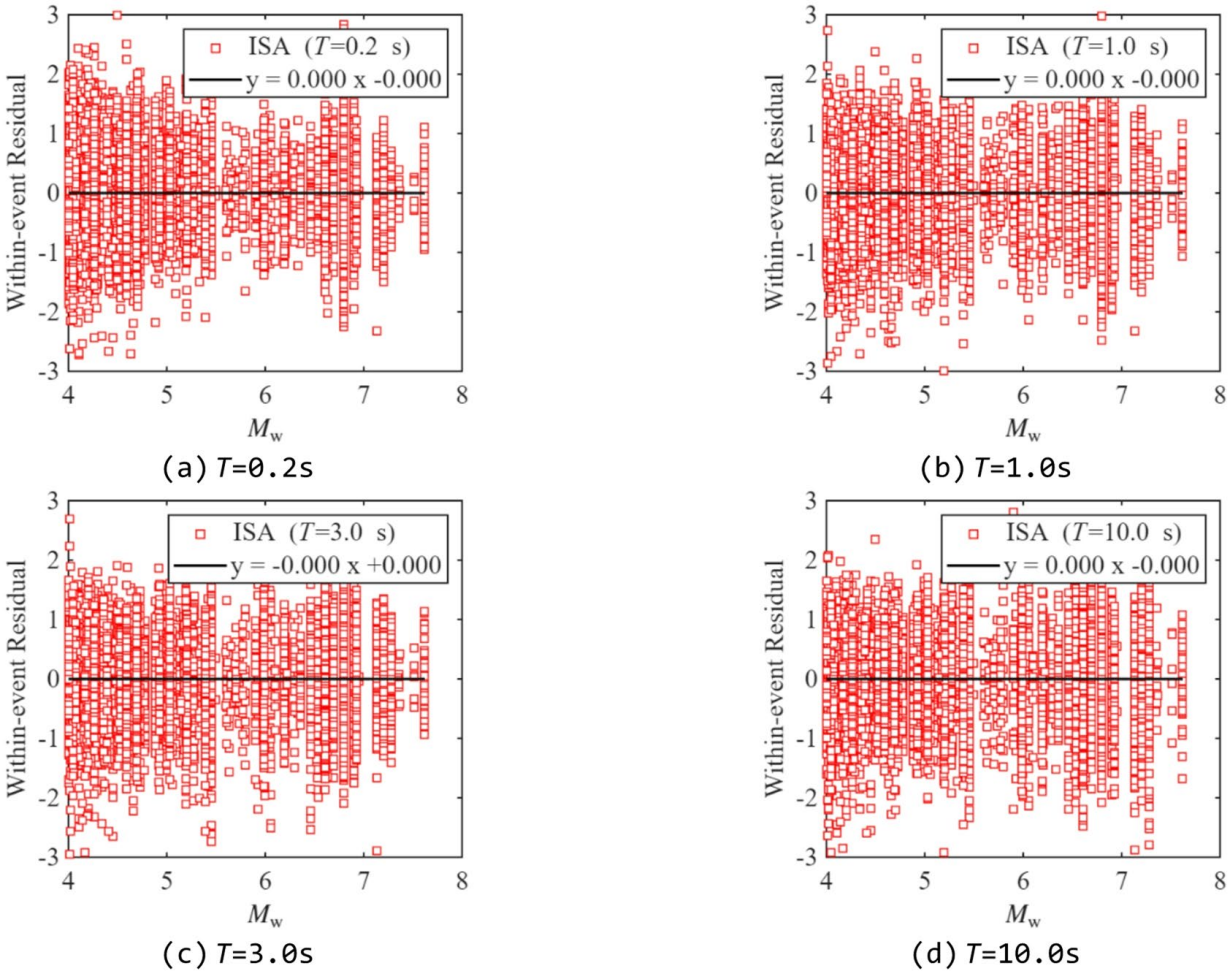
Distribution of within-event residuals of ISAs related to *M*_w_ (*R*_y_ =4).

**Fig. 15:**
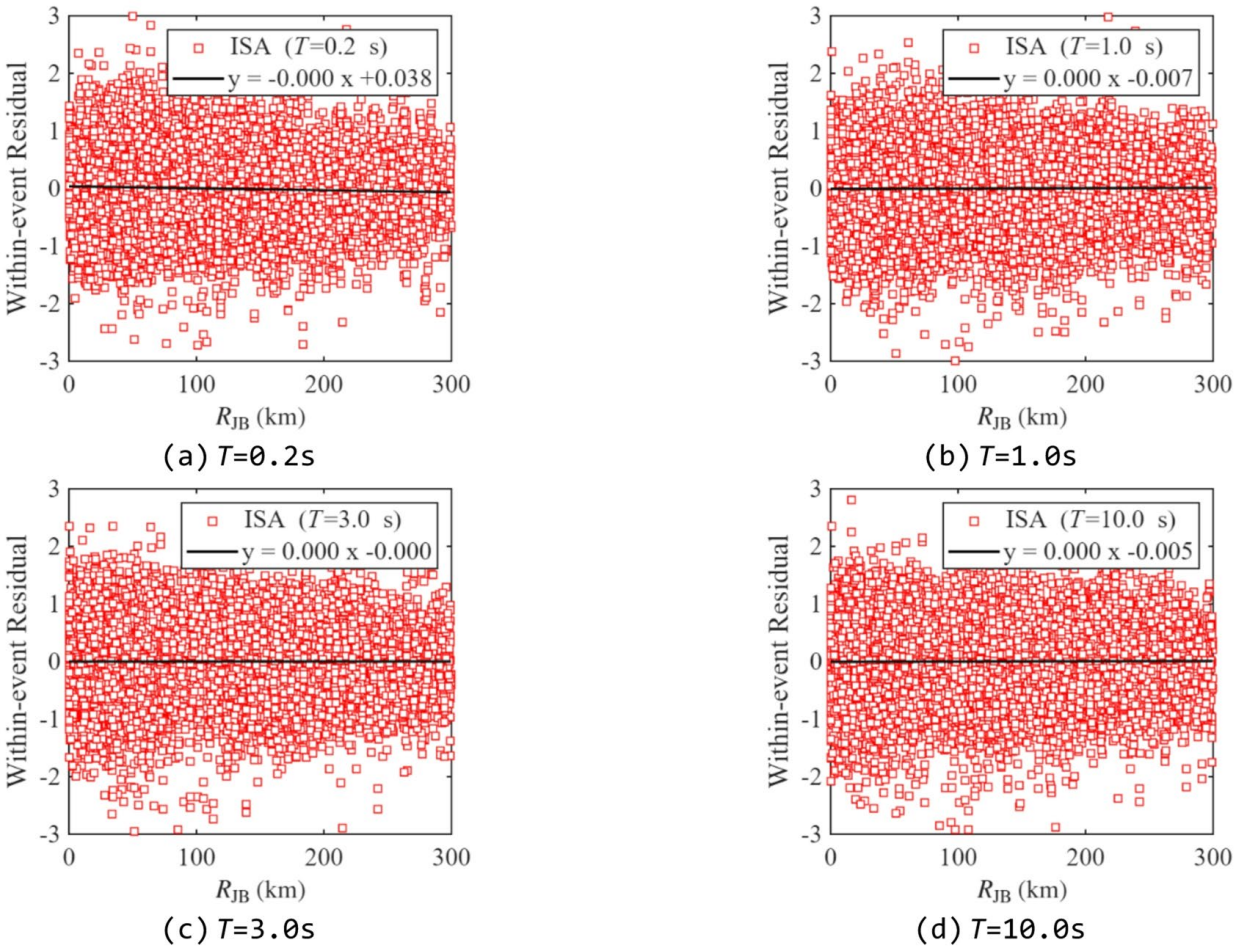
Distribution of within-event residuals of ISAs related to *R*_JB_ (*R*_y_ =4).

**Fig. 16:**
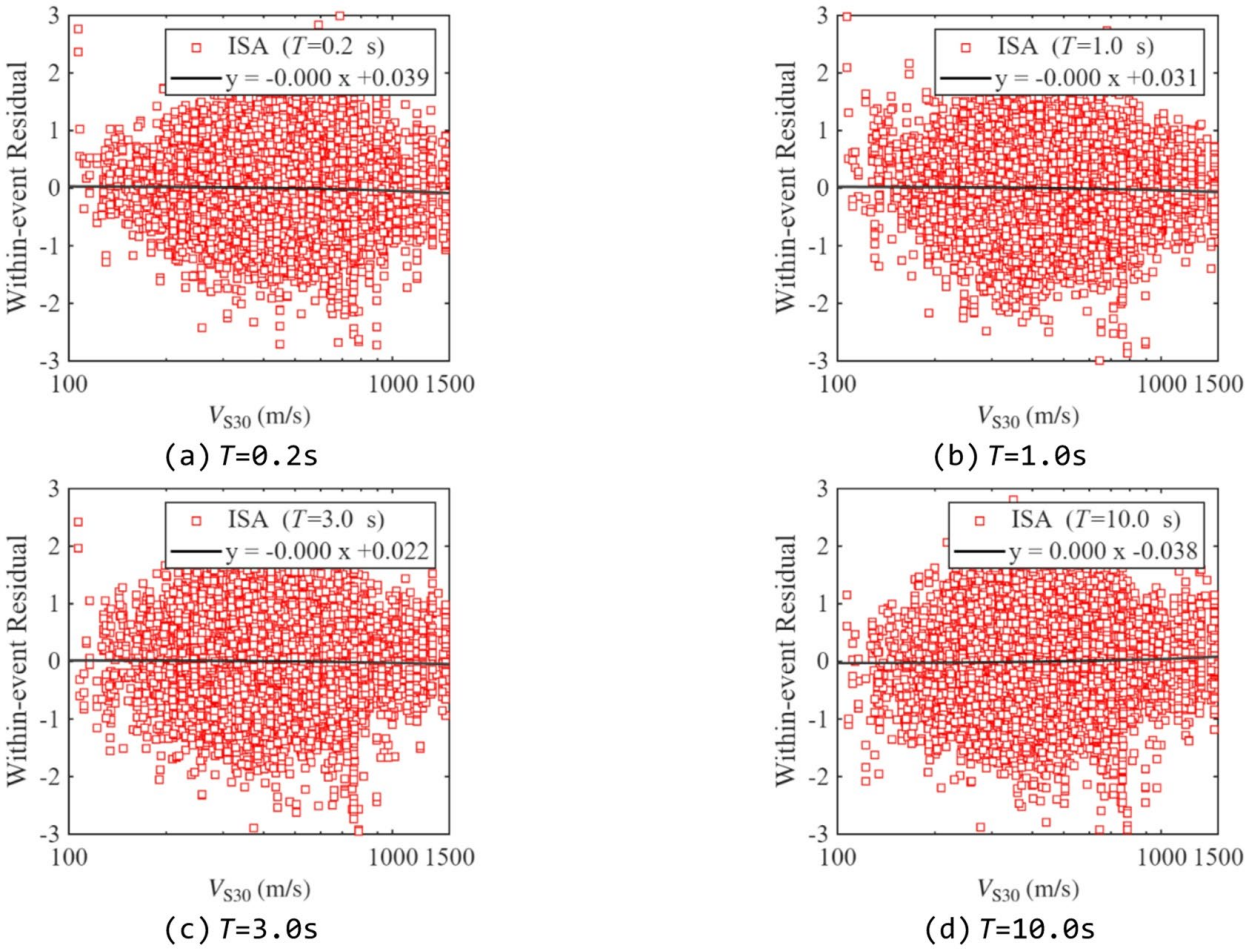
Distribution of within-event residuals of ISAs related to *V*_S30_ (*R*_y_=4).

Residual analysis was performed to evaluate the predictive performance of the proposed model, as it can reveal whether the model exhibits bias over specific ranges of the independent variables. Figures 13 and 14 show the between-event and within-event residual distributions of ISA at different periods relative to *M*_w_ for *R*_y_= 4. Figures 15 and 16 present the within-event residual distributions of ISA at different periods relative to *R*_JB_ and *V*_S30_. The gray shaded area indicates the 95% confidence interval of the trend.

As shown in Fig. 13, most between-event residuals are distributed within the range of [−1, 1] and remain consistent across different periods, indicating no significant systematic bias at the event level. However, a slight positive trend in residuals with increasing magnitude can be observed, indicating that the model may underestimate ISA for larger earthquakes and slightly overestimate it for smaller ones. The 95% confidence intervals are generally narrow, indicating stable estimates across most magnitude ranges. However, the interval widens for large earthquakes due to the limited number of records.

In Fig. 14-Fig. 16, most within-event residuals are distributed within [−2, 2], and their mean values do not exhibit any significant trend within the confidence interval, indicating negligible bias in the model with respect to *M*_w_, *V*_S30_, and *R*_JB_. Finally, comparing between-event and within-event residuals shows that within-event residuals are larger than between-event residuals, which is consistent with the findings from previous studies^23,48^.

To further quantify the aleatory variability implied by these residual patterns, the mean residual, between-event standard deviation (τ), within-event standard deviation (φ), and total standard deviation (σ) were computed for each period and are summarized in Table 5.

**Table 5:** Values of mean residual, τ, φ, and *σ* of test set for ISAs with different periods.

ISA	Mean residual	τ	φ	σ
ISA (*T* = 0.2s)	0.2025	0.6653	0.6892	0.9579
ISA (*T* = 1.0s)	0.1270	0.6889	0.6872	0.9730
ISA (*T* = 3.0s)	0.0980	0.7754	0.6982	1.0434
ISA (*T* = 10.0s)	0.2601	0.7629	0.7057	1.0392

### Result and validation of the XGBoost-based GMM

**Fig. 17:**
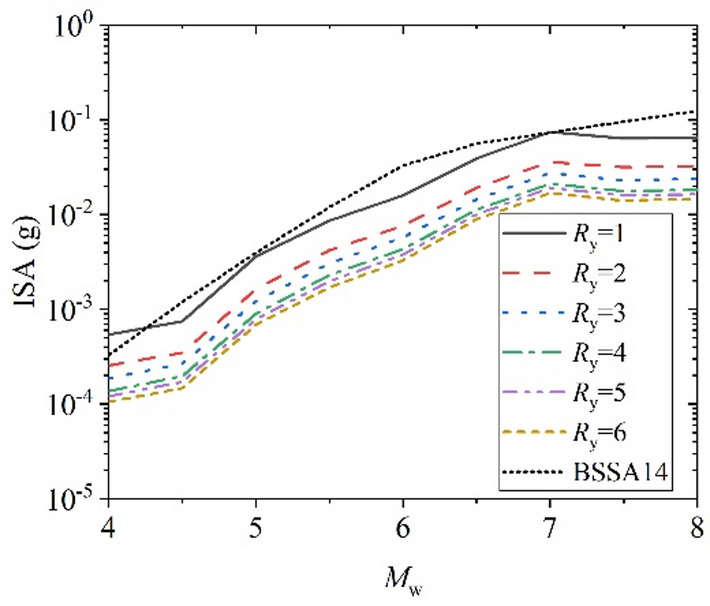
ISA versus *M*_w_ for various *R*_y_. (*T* = 1.0 s, *R*_JB_ = 30 km, and *V*_S30_ = 760 m/s).

Further evaluation was conducted on the proposed XGBoost-based GMM and compared with the classical empirical model BSSA14^4^ as a reference. Since BSSA14 is an elastic GMPE corresponding to *R*_y_ =1 and no established model currently exists for constant-strength inelastic response spectra, BSSA14 is adopted solely to examine the physical consistency of the proposed model in the elastic limit, rather than for predictive performance comparison. By varying one parameter at a time as the independent variable, the trends of ISA with different *R*_y_ were analyzed. Figure 17–Fig. 20 illustrates the relationships between ISA and *M*_w_, *T*, *R*_JB_, and *V*_S30_, and the prediction results of traditional BSSA14 model are also shown in each figure with same input parameters for comparison. Overall, ISA decreases with increasing of *R*_y_, indicating the reduction in seismic demand due to inelastic energy dissipation. As the SDOF structural inelastic response level increases, the extent of reduction in ISA diminishes with the increasing of *R*_y_.

**Fig. 18:**
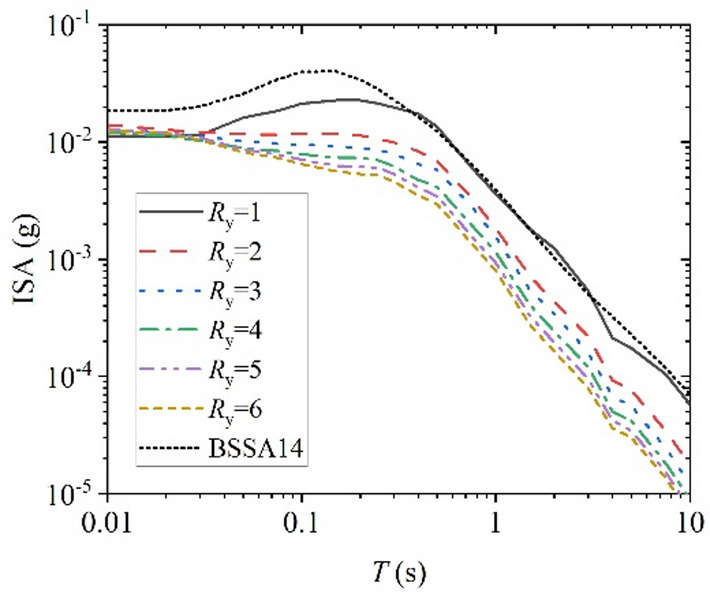
ISA versus period for various *R*_y_. (*M*_w_ = 5.0, *R*_JB_ = 30 km, and *V*_S30_ = 760 m/s).

Furthermore, Fig. 17 shows an increase trend of ISA with increasing *M*_w_, which indicates that in case of elastic case *R*_y_=1, the prediction results from this study’s model are essentially as same as those of BSSA14 traditional prediction model. Moreover, as the magnitude increases, the values of the ISA increase exponentially. Figure 18 reveals the variation of ISA with structural period, showing a marked decay at longer periods. It can be seen that the predicted results of this study are slightly smaller than those predicted by traditional prediction model BSSA14 in the short period range of 0.01–0.3 s, possibly due to differences in the representation of short-period frequency content and parameterization between the two models. The differences are not significant when the period is larger than 0.3s. Except for elastic case *R*_y_=1, as the period increases, the response spectra values decrease rapidly, which is consistent with the inelastic response spectra characteristics of ground motion.

**Fig. 19:**
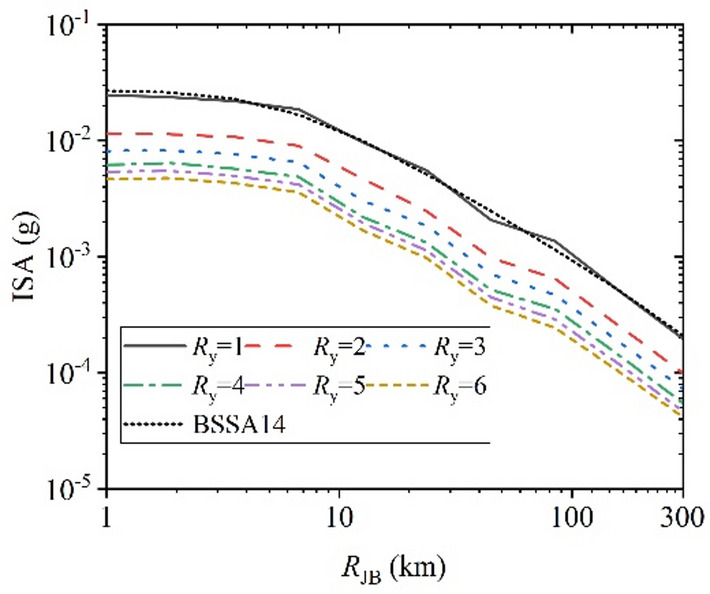
ISA versus *R*_JB_ for various *R*_y_. (*M*_w_ = 5.0, *T* = 1.0 s, and *V*_S30_ = 760 m/s).

**Fig. 20:**
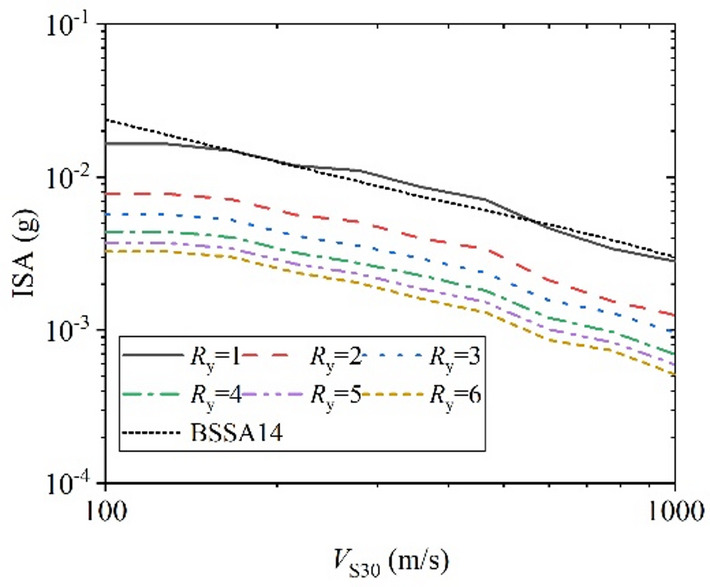
ISA versus *V*_S30_ for various *R*_y_. (*M*_w_ = 5.0, *T* = 1.0 s, and *R*_JB_ = 30 km).

Figure 19 shows that ISA decreases rapidly with increasing *R*_JB_, reflecting the controlling effect of the propagation path. When the fault distance is small (*R*_JB_ less than 8 km), it exhibits a certain near-field saturation phenomena, which is consistent with the ground motion parameters’ attenuation laws. Figure 20 indicates that ISA decreases with increasing *V*_S30_, highlighting the influence of site condition effects. As shown in Fig. 20, with the increasing of *V*_S30_, that is, as the site becomes harder, the ISA values decrease, which is related to the well-known amplification effect of soft sites on ground motion. For the elastic case *R*_y_=1, the predictions from this study are almost identical to the traditional BSSA14 model.

In summary, the variation of ISA is jointly determined by the coupling of source characteristics, propagation path, site conditions, and structural dynamic properties. The consistency of the predicted trends with those of the empirical BSSA14 further validates the physical soundness of the proposed approach.

## Conclusions

This study proposes an XGBoost-based GMM for predicting the inelastic spectral acceleration (ISA), which is the most important type of the inelastic response spectra (IRS). The investigation focuses on two aspects: the superiority of the predictions and the interpretability of the input features. To this end, 15,512 ground motion records were selected from the NGA-West2 database, and the ISA at constant-strength were computed for each record. The XGBoost model was trained, validated, and tested with an 8:1:1 split, and multiple performance metrics, along with residual analyses, were employed to assess the predictive performance. The main conclusions are summarized as follows:

1. The proposed XGBoost-based GMM achieves high *R*² values and low RMSE and MAE across the training, validation, and testing datasets, demonstrating strong predictive capability for ISA. The residual analysis with most inter-event residuals contained within [− 0.5, 0.5] and most intra-event residuals within [− 2, 2], further confirms the model’s robustness.
2. The model accurately captures the fundamental physical relationships governing ISA, including the effects of structural period, strength reduction factor, magnitude, distance, and site conditions. The interpretability analysis confirms that the learned feature contributions align with established seismological and structural engineering principles, enhancing the transparency and credibility of the model.
3. Trend-comparison analyses confirm that the XGBoost-based GMM captures the coupled effects of source, path, site, and structural period in a manner consistent with the established BSSA14 model, while additionally providing accurate predictions across a wide range of inelasticity levels. This demonstrates that the model effectively extends traditional GMM concepts into the inelastic domain.

Finally, this study has several limitations. In particular, the ISA database is generated using a single hysteretic model, and alternative hysteretic behaviors, such as those represented by the Modified Clough (MC) model and the Pinching Hysteresis (PH) model, are not considered. Different hysteretic models may influence the absolute level of inelastic spectral demands. Future studies could address this limitation by incorporating multiple hysteretic models and constructing larger and more comprehensive databases, thereby further improving the generality and applicability of the proposed model. In addition, the proposed model is intended primarily for interpolation within the parameter space covered by the training data. Extrapolation beyond this range, particularly for extreme earthquake magnitudes, spectral periods, or site conditions, may introduce increased uncertainty.

## Data Availability

The strong ground motion data used in this study are available at https:/ngawest2.berkeley.edu.

## Code availability

The code developed by the authors has been uploaded to GitHub (https://github.com/gongyx2423/XGBoost-based-GMM). The readers could download the code from the website.
